# Exploring the regulatory role of lncRNA in cancer immunity

**DOI:** 10.3389/fonc.2023.1191913

**Published:** 2023-08-10

**Authors:** Dan-ting Zhan, Hong-chun Xian

**Affiliations:** ^1^ Department of Prosthodontics, Affiliated Stomatological Hospital of Southwest Medical University, Luzhou, China; ^2^ Department of Plastic and Maxillofacial Surgery, The Second Affiliated Hospital of Chongqing Medical University, Chongqing, China

**Keywords:** lncRNA, cancer immunity, cancer, immune evasion, tumor microenvironment

## Abstract

Imbalanced immune homeostasis in cancer microenvironment is a hallmark of cancer. Increasing evidence demonstrated that long non-coding RNAs (lncRNAs) have emerged as key regulatory molecules in directly blocking the cancer immunity cycle, apart from activating negative regulatory pathways for restraining tumor immunity. lncRNAs reshape the tumor microenvironment via the recruitment and activation of innate and adaptive lymphoid cells. In this review, we summarized the versatile mechanisms of lncRNAs implicated in cancer immunity cycle, including the inhibition of antitumor T cell activation, blockade of effector T cell recruitment, disruption of T cell homing, recruitment of immunosuppressive cells, and inducing an imbalance between antitumor effector cells (cytotoxic T lymphocytes, M1 macrophages, and T helper type 1 cells) versus immunosuppressive cells (M2 macrophages, T helper type 2 cells, myeloid derived suppressor cells, and regulatory T cells) that infiltrate in the tumor. As such, we would highlight the potential of lncRNAs as novel targets for immunotherapy.

## 1 Introduction

long non-coding RNAs (lncRNAs) have been defined as a type of transcript longer than 200 nucleotides in length without protein-coding capacity (1, 2) and featured as a spliced structure, with poorly conserved sequences and a polyadenylated tail (3). In the nucleus, lncRNAs act as the signal, guide, decoy, and scaffold to modulate chromatin organizations and transcriptional regulation (4). In the cytoplasm, lncRNAs modulate mRNA stability, translation, and post-translational modification (5). lncRNAs have emerged with a vital role in regulating various biological processes in cancer, such as proliferation, differentiation, metabolism, and immune response (6–8). Aberrantly expressed lncRNAs exhibit a tissue-specific manner in cancer, which could be biomarkers for diagnosis, prognostic prediction, as well as potential therapeutic targets (9–11). lncRNAs have been especially shown to be important in the context of anticancer immunity and immune evasion.

A dysfunctional immune status in the tumor microenvironment is a hallmark of cancer (12), and the imbalance of the dynamic immune state plays an essential role in the occurrence and progression of cancer. The sequential procedures featured with generating an immune response to effectively destruct cancer cells have been defined as the cancer immunity cycle (13). The generation of cancer immunity begins with the capture of tumor-specific antigens from dying cancer cells and then presents in the context of major histocompatibility complex (MHC)-I and MHC-II molecules by dendritic cells (DCs) to T cells, resulting in T cell priming and activation. Then, the activated T cells traffic back and infiltrate into the tumor, culminating in targeted killing of the cancer cell via recognition-specific cognate antigen on cancer cells—that is, the cancer immunity cycle contains the release and presentation of cancer antigens, priming and activation of effector T cell responses, trafficking and infiltration of T cells into tumors, recognition of cancer cells by T cells, and eradication of cancer cells (14). Understanding the role of lncRNAs in the processes involved in immune suppression and overpowering the negative feedback mechanisms of the cancer immunity cycle is crucial for new therapeutic strategies.

Recently, an increasing number of studies have demonstrated that lncRNAs act as a master mediator in immune escape, the counterbalance of an inhibitory and stimulative immune microenvironment. Here, according to each stage of the cancer immunity cycle, we reviewed the role of lncRNAs in coordinating antitumor responses and exquisitely elucidated their mechanisms of orchestrating cancer immunity through the expansion, function, differentiation, and balance of pro- and anti-tumor leukocytes including dendritic cells, regular T cells, cytotoxic T lymphocyte, helper T cells, tumor-associated macrophages, and myeloid-derived suppressor cells. A literature search was conducted using the Medline database, covering the period from January 2014 to March 2023. The following medical subject headings (MeSH) terms and keywords are listed as follows: (‘vascular endothelial cells’ or ‘chemokines’ or ‘dendritic cells’ or ‘tumor-associated macrophages’ or ‘tumor-associated neutrophils’ or ‘myeloid-derived suppressor cells’ or ‘regulatory T cells’ or ‘T helper cell’ or ‘natural killer cells’ or ‘cytotoxic T lymphatic’) AND (‘long non-coding RNA’ or ‘lncRNA’ or ‘ncRNA’). Then, the potential predictive value of lncRNAs for immunotherapy response was discussed, aiming at screening potential immunotherapy targets for cancer.

## 2 lncRNAs and antigen presentation deficiency

Tumors often disturb normal DC activation and maturation to establish tumor tolerance (15–17). Immunosuppressive cytokines deriving from tumor contexture induce DCs to exhibit an immunosuppressive phenotype that mediates tumor tolerance, thereby restraining T cell priming (15, 18).

**Figure 1:**
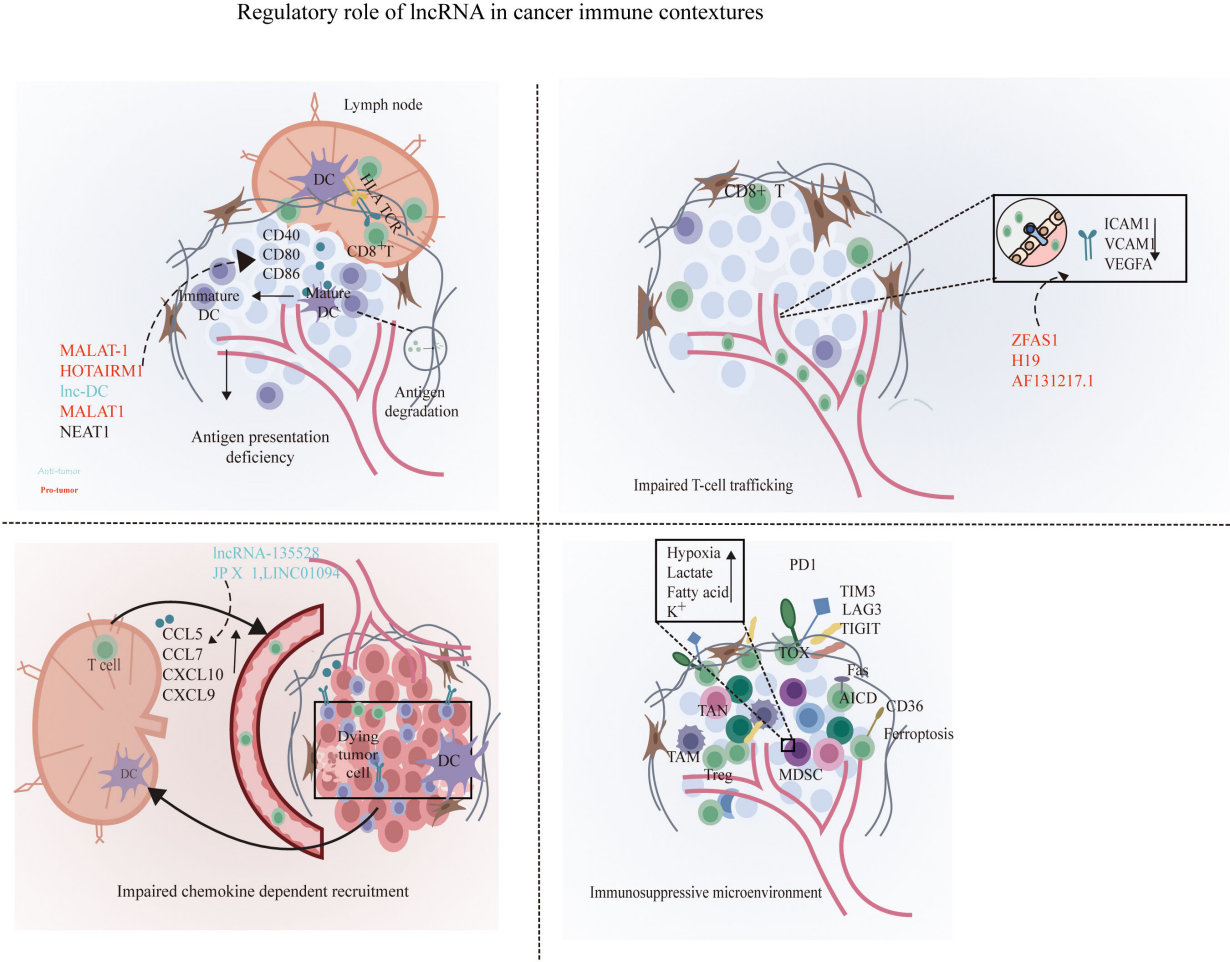
Disruption of the cancer immunity cycle by lncRNAs. lncRNAs can inhibit immune cells at multiple steps in the cancer immunity cycle. In the stage of T cell priming and activation, lncRNA such as MALAT1, lnc-DC, and NEAT1 are associated with the disruption of the maturation of DCs and suppress dendritic cell costimulatory abilities, leading to reduced cancer antigen presentation and increased anti-inflammatory cytokines such as IL-10. Next, lncRNA XIST, LNMAT1, lnc-sox5, lnc-BM, and lncRNA-135528 are involved in the blockade of immune cell recruitment through regulating the expression and secretion of chemokines such as CCL2, CCL5, CSCF1, CXCL9/10, and IL-6/23. Before effector T migrates into tumor to eliminate cancer cells, lncRNAs ZFAS1, H19, and HOTAIRM1 facilitate immune cell traffic across the vascular endothelial barrier so as to infiltrate into solid tumor. In the elimination phase, tumor cells and immune-suppressive immune cells were exploited to weaken and deactivate the cytotoxicity of CTLs. At this stage, lncRNAs can suppress CD8+ T cell effector function by skewing M1/M2 and Th1/Th2 polarization and indirectly elicit Tregs and MDSCs to inhibit CD8+ T cells and prevent the elimination of cancer cells. At the final step in the cancer immunity cycle, lncRNA such as SNHG14, NKILA, and lnc-Tim3 can directly inhibit CD8+ T cell effector function through the induction of exhausted phenotype and activation-induced cell death of effector T cells, resulting in impaired efficient and durable CTL responses. By shaping a repressive immune microenvironment, tumor cells obtain immune escape.

Accumulated studies demonstrated that lncRNAs could facilitate immune evasion through inducing a tolerogenic and immunosuppressive DC phenotype (**Figure 1**). lncRNA MALAT-1 promoted colon cancer progression by inducing the dendritic cell-mediated immune tolerance (19). Consistently, inhibition of MALAT1 was also identified to induce a tolerogenic DC phenotype that secrete increased immunosuppressive molecules so as to induce T cell anergy and promote Treg generation through IDO signaling (20). In addition, HOTAIRM1, a widely researched tumor-associated lncRNA in endometrial cancer (21), ovarian cancer (22), and colorectal cancer (23), promoted DC maturation by competitively bonding to miR-3960 and prevented it from targeting the DC maturation markers such as CD40, CD80, and CD86 (24). Another similar oncogenic lncRNA, lnc-DC, also played a vital role in dendritic cell maturation, and the knockdown of lnc-DC impaired DC maturation and reduced the ability of DCs to efficiently prime T cells. lnc-DC mediates these effects by activating the transcription factor signal transducer and transcriptional activator 3 (STAT3) that regulates DC differentiation and promoting STAT3 tyrosine-705 phosphorylation (25). Moreover, nuclear enriched abundant transcript 1 (NEAT1) could also induce a tolerogenic DC phenotype by sponging miR-3076-3p, thus facilitating the induction of tolerogenic phenotype in DCs via NLRP3 inflammasome, which was required for the differentiation of DCs (26). The capacity of lncRNAs to balance the tumor microenvironment (TME) and their immunosuppressive properties to disturb the cancer immunity cycle is needed to appropriately activate DCs. lncRNAs could altogether reduce the number of cells capable of recognizing and eradicating neoplastic-transformed cells via modulation of the activation and maturation of DCs. Hence, these findings authenticate the pivotal roles of lncRNAs in antigen presentation, and manipulating lncRNAs may contribute to reversing the antigen presentation deficiency.

## 3 lncRNAs and chemokine-dependent recruitment

After priming and activation, T cells are recruited through cytokines, chemokines, and adhesion molecules generated within the TME. Tumors suppress the recruitment of T cells via the disruption of normal T cell-attracting chemokine elaboration, such as chemokine (C-C motif) ligand 2 (CCL2), CCL3, CCL4, CCL5, chemokine (C-X-C motif) ligand 9 (CXCL9), and CXCL10 (27, 28). Among them, CXCL9 and CXCL10 are essential for the recruitment of CD8+ T cells through the corresponding receptor CXCR3 (29). lncRNA-135528 has been demonstrated to disrupt the CXCL9/CXCL10-dependent infiltration of T cells through the activation of the JAK/STAT pathway, thus inhibiting glioma progression (30). lncRNA JP X functions as a sponge of miR-378g to upregulate CCL5 (31), which could promote the recruitment of CD8+ T cells into tumors (32, 33). Hence, lncRNAs could mediate the deregulation of effector T cell recruitment through aberrantly unleashing the amount and altering the activities of expressed chemokines.

Apart from mediating the recruitment of antitumor T cell subsets, lncRNA could orchestrate the crosstalk between cancer cells and immune cells via the release of cancer-derived chemokines such as CC-chemokine ligand 2 (CCL2), vascular endothelial growth factor A (VEGFA), fibroblast growth factor (FGF), hepatocyte growth factor (HGF), CCL5, CCL7, and CCL22 to enhance immunosuppressive lymphocyte recruitment and infiltration such as TAMs, MDSCs, and Tregs (27, 34). Lymph node metastasis-associated transcript 1 (LNMAT1) was significantly upregulated in bladder cancer with lymph node metastasis and is prognostic of overall survival in patients with bladder cancer. Mechanistically, LNMAT1 recruited hnRNPL-mediated H3 lysine 4 trimethylation (H3K4me3) to the CCL2 promoter and epigenetically activated CCL2 expression to recruit TAMs in the tumor microenvironment (35). lnc-BM (a lncRNA related to breast cancer brain metastasis) in breast cancer cells binds to JAK2 and modulates its kinase activity through the lnc-BM/JAK2/STAT3/ICAM1 pathway and promotes the release of chemokine CCL2 to attract macrophages, ultimately elevating the metastatic potential of breast cancer cells to the brain (36). Moreover, the overexpression of JHDM1D-AS1 in pancreatic cancer cells could raise the infiltration of macrophage through promoting the secretion of tumor-derived chemokines such as HGF and FGF1 (37). LINC01094 increased the expression of CCL7 in lung adenocarcinoma by binding to the transcription factor SPI1 of CCL7 and promoting its translocation into the nucleus, thus increasing the degree of infiltration of macrophages in lung adenocarcinoma (38). lnc-sox5 could increase the infiltration of Treg cells by promoting the level of soluble chemokine indoleamine 2,3-dioxygenase 1 (IDO1), further stimulating the progression of colorectal carcinogenesis (39). lncRNA XIST could accelerate colorectal cancer tumorigenesis and progression through functioning as a ceRNA to sponge miR-133a-3p to increase the serum level of IL-1, IL-6, and TNF-α, resulting in massive infiltration of MDSCs and macrophages (40).

## 4 lncRNAs and T cell trafficking

After priming in tumor-draining lymph nodes, trans-migration of the activated T cells through the vascular endothelium into the tumor is the rate-limiting step for effective tumor immunity (41). Tumors can modulate the endothelial barrier through tumor-derived immunosuppressive and proangiogenic cytokines such as TNF-α, VEGF, interleukin 10 (IL-10), and prostaglandin E2 (PGE2) and inhibit the expression of adhesion molecules such as vascular cell adhesion molecule-1 (VCAM1) and intercellular adhesion molecule-1 (ICAM1) on endothelium that propitiate the adhesion of T cells or induce the expression of molecules that prompt the cell eradication of effecter T cells (42, 43).

lncRNA ZFAS1 was identified to function as an endogenous sponge to upregulate VEGFA via competitively binding to miR-150-5p (44). Consistent with this, overexpressed lncRNA ZFAS1 may induce an anergy phenotype of the endothelium and impair blood vessel permeability, thus upregulated VEGF signaling to promote the infiltration of effector T cells into the tumor microenvironment and promote colorectal carcinoma progression. On the other hand, the extravasation of leukocytes was elicited by gradients of selectins expressed on the endothelial cell surface, such as intercellular adhesion molecule 1 (ICAM-1) and vascular cell adhesion molecule 1(VCAM). lncRNA H19 which widely participated in cancer progression (45) had also been identified to suppress the expression of ICAM-1 and VCAM-1 in endothelial cells through repressing the phosphorylation of STAT3 (46). lncRNA AF131217.1 could modulate the expression of adhesion molecules in endothelial cells, and the knockdown of AF131217.1 promoted ICAM-1 and VCAM-1 expression (47). Several other studies elucidated that lncRNAs could be involved in promoting the secretion of ICAM-1 and VCAM-1 in endothelial cells, such as LINC00341 (48), DLGAP1-AS1 (49), lncRNA UCA1 (50), and AK094457 (51). These findings suggested that lncRNAs may mediate endothelium signaling to affect T cell trafficking in cancer immunity microenvironment.

## 5 lncRNAs and local immunosuppressive tumor microenvironment

**Figure 2:**
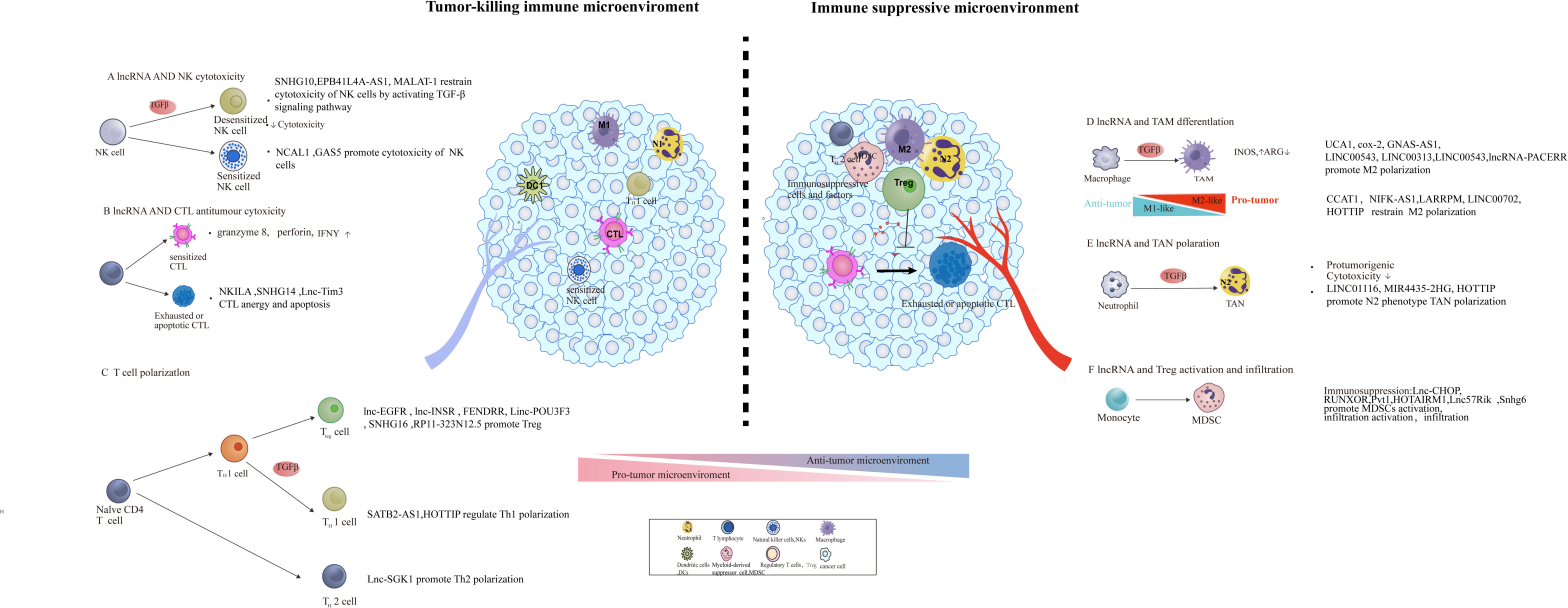
lncRNA involved in the construction of an immunosuppressive tumor microenvironment through modulating the function, differentiation, and infiltration of pro- and anti-tumoral leukocytes. The regulatory role of lncRNA in tumor microenvironment remodeling, including the activation of immunosuppressive cells and factors [e.g., MDSCs, tumor-associated macrophage (TAM) subsets] and initiation of abnormal antitumor immune cells (e.g., DC, NK, and T cells) and Tregs.

Upon the effector T cells navigating the tumor stroma to target tumor cells, immunosuppressive leukocyte populations such as tumor-associated macrophages (TAMs), myeloid-derived suppressor cells (MDSCs), tumor-associated neutrophils (TANs), regular T cells (Tregs), T helper 2 cells(Th2) actively recruited and underwent local expansion to establish an immune tolerance barrier that effectively suppresses the function of antitumor immune cells such as cytotoxic T lymphocytes (CTLs) and T helper 1 cells (Th1) effector cells (28, 52–55). Accumulating evidence indicates the role of lncRNAs in the modulation of the balance between an immunosuppressive microenvironment and a more favorable environment for T cell function. lncRNAs can also engage in the phenotype switch of cells, such as helper T cell, and macrophage, MDSC which can contribute to the establishment of immunosuppressive TME (**Figure 2**).

### 5.1 TAMs

TAMs are an essential immunosuppressive component and display an ability to suppress T cell recruitment and function (56). TAMs display heterogeneity and could differentiate into two primary subtypes, M1 and M2 type (57). M1 macrophage generally promotes tumoricidal activities, while M2 macrophage is critical for immunological tolerance (58, 59). A number of recent studies exemplified that lncRNAs were involved in macrophage polarization by regulating proinflammatory gene expression, such as MALAT-1 and UCA1. Upregulated MALAT-1 in TAMs isolated from thyroid cancer were able to skew macrophages towards M2 polarization, while the knockdown of MALAT-1 could promote macrophage repolarization from the M2 phenotype to the M1 phenotype and further inhibited thyroid cancer cell migration and invasion (60). Similarly, lncRNA UCA1 contributes to tumor growth by triggering the M2 polarization of TAMs while decreasing the infiltration of M1 cells. The knockdown of UCA1 inhibited reversing this polarization trend and impaired the tumor growth and invasion (61). Moreover, in triple-negative breast cancer, lncRNA HOTAIR and MALAT1 play a synergistic role in promoting M2-like polarization of macrophages, and the simultaneous knockout of HOTAIR and MALAT1 can increase the expression levels of M1-like macrophage markers CD80 and MSLN (62).

A series of experiments has been performed to identify the regulatory role of lncRNA in M2 polarization. On the one hand, lncRNA could stimulate cancer progression by facilitating M2 polarization—for instance, GNAS-AS1 expression was reported to markedly increase in TAMs isolated from non-small cell lung cancer cells. The knockdown of GNAS-AS1 could suppress M2 macrophage polarization, thus reducing the proliferation, migration, and invasion of non-small cell lung cancer through sponging miR-4319 (63). Another illustration is lincRNA COX-2, whose expression level in M1 was elevated than that in M2 in hepatocellular carcinoma (HCC). Meanwhile, the knockdown of lincRNA COX-2 facilitated M2 polarization and repressed HCC cell proliferation, apoptosis, metastasis, EMT, angiogenesis, and tumor formation *in vivo (* 64). LINC00543 activates CCL2-mediated M2 polarization through the pre-miR-506-3p/FOXQ1 axis and ultimately initiates the metastasis of colorectal cancer (65). In ovarian cancer, lncRNA Xist could promote the polarization of M2-like macrophages by competitively binding miR-101 to upregulate the expression levels of CEBP-α and KLF6 (66). In the cytoplasm, lncRNA-PACERR improves the mRNA stability of KLF12 and c-myc by binding IGF2BP2, thereby promoting the differentiation of M2-like macrophages to promote the progression of pancreatic ductal carcinoma. At the same time, KLF12 can enter the nucleus and bind to the lncRNA-PACERR promoter region, promoting the acetylation of histones in this region and thereby promoting the transcription of lncRNA-PACERR and amplifying this positive feedback loop. In addition to acting as a molecular sponge and mRNA stability regulator, lncRNAs can also affect macrophage differentiation at the epigenetic level—for instance, lncRNA-PACERR binds and guides the transcription factor CTCF and histone modification complex p300 to the PTGS2 promoter region in the nucleus, promotes the acetylation of histones in this region, improves the expression level of PTGS2, and thus initiates the differentiation of monocytes into M2-like macrophages (67).

On the other hand, lncRNAs withheld cancer progression through restraining M2 polarization—for example, colon cancer-associated transcript-1 (CCAT1) could block M2 macrophage polarization by modulating miR-148a and PKC-ζ interaction, thereby halting the cell migration of prostate cancer (68). Analogously, NIFK-AS1 could inhibit the M2-like polarization of macrophages through preventing miR-146a from targeting Notch1, thus inhibiting the proliferation, migration, and invasion of endometrial cancer (69). In the context of lung adenocarcinoma, lncRNA LARRPM restricts CSF1-mediated M2 macrophage polarization by guiding TET1 to the CSF1 promoter; as a result, DNA methylation of CSF1 promoter increases, and the transcription is repressed (70).

The low expression of LINC00702 in bladder cancer can inhibit the carcinogenic effect of M2-like macrophages by promoting DUSP1 transcription through recruiting JUND to its promoter (71). In addition to directly inhibiting M2-like polarization, lncRNA can also promote the differentiation of macrophages in tissues to M1-like differentiation, thereby indirectly inhibiting the M2-like polarization of macrophages. In head and neck squamous cell carcinoma, lncRNA HOTTIP encapsulated in the exosomes of M1-like macrophages induces monocytes in the tissue to differentiate into M1-like macrophages, thereby inhibiting tumor progression. In the cytoplasm, HOTTIP acts as a molecular sponge to desorb miR-19a-3p and miR-19b-3p, thereby activating the TLR5/NF-κB signaling pathway (72).

Tumor cells and macrophages exchange lncRNAs by secreting exosomes to influence and transmit information to each other. Hepatocellular carcinoma cell-derived lncRNA miR4458HG is packaged and sent in forms of exosomes to exert its oncogenic role by promoting the M2-type polarization macrophage via increasing ARG1 expression (73). Similarly, non-small cell lung cancer-derived exosomal LINC00313 acts as miR-135a-3p sponge to induce M2 macrophage differentiation by upregulating STAT6 (74). The communication between tumor cells and macrophages is bidirectional, and macrophages can also influence the biological behavior of tumor cells by secreting exosomes. In bladder cancer, M2-like macrophage exosome-derived H19 could enhance ULK1 stabilization through alleviating the K48-linked polyubiquitination of ULK1, sequentially facilitating the autophagy of bladder cancer cell (75). Osteosarcoma cells transmit ELNF1-AS1 in the form of exosomes to macrophages. Acting as molecular sponges in macrophages, ELNF1-AS1 is capable of binding miR-138-5p and miR-1291 simultaneously and decomposing the inhibitory effect of miR-1291 on CREB1 expression. It makes M2-like macrophages become the dominant cell population and shapes a tumor microenvironment that promotes cancer (76). Macrophage-derived exosomal lncRNA LIFR-AS1 promotes osteosarcoma cell progression via the miR-29a/NFIA axis (77). lncRNA CRNDE in TAM-derived exosomes could improve the resistance of gastric cancer cells to cisplatin (78).

Apparently, lncRNAs played an important role in maintaining the equilibrium between M1 and M2 types that promoted or suppressed anticancer immunity.

### 5.2 TANs

Tumor-associated neutrophils (TANs) are important components in the tumor microenvironment. Analogous to the M1/M2 paradigm of macrophages, TANs alternatively polarize into phenotype N1 (antitumorigenic) and phenotype N2 (pro-tumorigenic) (79). lncRNAs plays a significant role in the determination of the neutrophils’ phenotype by shifting the equilibrium from an N1 phenotype toward a more permissive N2 phenotype (80).

The accumulated TAN in the tumor microenvironment produced a variety of cytokines to inhibit T cell activation, reconstruct tumor extracellular matrix, and stimulate angiogenesis in such a way to promote tumor progression (81). It has been demonstrated that lncRNAs participate in modifying the immune microenvironment by influencing neutrophil homing and infiltration. Upregulated LINC01116 can increase neutrophil recruitment by stimulating the production and secretion of IL-1β via recruiting DDX5 to the IL-1β promoter and ultimately facilitating glioma proliferation (82). MIR4435-2HG exerts its immunogenic effects in colorectal cancer; in the context of MIR4435-2HG knockdown, antitumorigenic phenotype N1 infiltration increased (83). In the context of ovarian cancer, lncRNA HOTTIP has been verified to endow neutrophil-induced effector T cell apoptosis by boosting PD-L1 expression in neutrophils via activating the IL-6 signaling pathway by means of binding to c-jun in the nucleus (84).

### 5.3 NK cells

NKs employ death receptor-mediated apoptosis or directly release perforin and granzyme-mediated cytotoxicity to eliminate tumor cells, in such a way acting as the body’s first line of defense (85). The cytotoxic activity of NK cells can also be regulated by long non-coding RNA. Within the colorectal cancer framework, upregulated lncRNA SNHG10 suppressed the viability and cytotoxicity of NK cells, driven by upregulating INHBC expression, causing the activation of the TGF-β signaling pathway (86). Contrary to SNHG10, accumulation of lncRNA NCAL1 fuels the cytotoxicity of NK cells that target tumor cells through increasing the H3K4me3 and H3K27ac levels in the Gab2 promoter, ultimately boosting the PI3K-AKT pathway mediated by Gab2 (87). lncRNA GAS5 restrain the growth and metastasis of liver cancer through motivating the killing effect of NK cells via directly sponging miR-544 to target RUNX3. As proof, lncRNA GAS5 can also enhance the tumor elimination ability of NK target gastric cancer by promoting the secretion of IFN-γ and TNF-α via regulating miR-18a (88). Moreover, in neuroblastoma, the lncRNA EPB41L4A-AS1 present in exosomes can be transmitted in subsets of NK cells, disrupting the immune elimination function of NK cells by damaging the glycolysis process of NK cells, thereby promoting tumor progression (89). MALAT-1 has been shown to relieve the inhibitory effect of miR-34a on the expression of PD-L1 and B7-H4 on the surface immune checkpoints of triple-negative breast cancer cells, which can induce the apoptosis of NKs and cytotoxic T cells in the tumor microenvironment. Thus, MALAT-1 is able to shape an immunosuppressive microenvironment by inhibiting immune effector cells and giving tumor cells the ability to evade immune elimination (90).

### 5.4 MDSCs

MDSCs are a heterogeneous immunosuppressive cell population of immature myeloid cells in the tumor microenvironment that express CD11b and GR1 (91, 92). In cancer progression, MDSCs contribute to the suppression of T cells and regulatory T cells through the production of suppressive cytokines such as arginase 1 (Arg1), reactive oxygen species (ROS), and iNOS (93, 94). lncRNAs such as lnc-chop, Pvt1, RUNXOR, Lnc57Rik, and HOTAIRM1had been reported to facilitate tumor progression through promoting MDSC differentiation and the secretion of immunosuppressive cytokines. The lnc-chop interacted with CHOP and dissociated the C/EBPβ isoform LIP from LAP so as to improve the activation of LAP, which initiated the expression of immunosuppressive genes such as Arg-1, NOS2, NOX2, and COX2 (95). Similarly, Lnc57Rik can not only bind with the C/EBPβ isoform LAP to activate C/EBPβ but also with the WDR5 that enables the enrichment of H3K4 on the promoter regions of Arg-1, NOS2, NOX2, and COX2, eventually resulting in their transcriptional activation in tumor (96). In esophageal cancer, Lnc-17Rik could act as decoy to dissociate C/EBPβ isoform LAP to activate C/EBPβ and as a scaffold to guide WDR5 transfer to the promoter region of MDSC-specific molecules, ultimately promoting the immunosuppressive function of MDSCs (97). The knockdown of Pvt1 significantly suppressed MDSC-mediated immunosuppression on T cells *in vitro* by decreasing the level of ROS and Arg1 (98). In addition, lncRNA RUNXOR could stimulate the differentiation and immunosuppression function of MDSCs by targeting RUNX1 in lung cancer. lncRNA RUNXOR knockdown inhibited the function of MDSCs through decreasing the expression of immunosuppressive molecules such as Arg1 in MDSCs (99). In contrast to RUNXOR, lncRNA HOTAIRM1 exhibited to suppress MDSC differentiation and downregulated the expression of suppressive molecules released by MDSCs in lung cancer (20). Mechanistically, HOTAIRM1 could impair the antitumor immune response by MDSCs and delay tumor progression though increasing the expression of HOXA1 in MDSCs, which promotes Arg1 secretion. In addition, lncRNA Snhg6 could facilitate the differentiation of MDSCs by inhibiting the expression of EZH2 by means of the ubiquitination pathway in lung cancer (100). These findings suggested that lncRNAs could alter the production of immunosuppressive molecules in MDSCs, such as Arg1 and ROS.

### 5.5 Tregs

Tregs exert influence at multiple steps of the cancer immunity cycle and play a prominent role in the regulation of antitumor immunity. Effective immune priming and activation are established by the relative ratio of T effector cells and T regulatory (Treg) cells. Several lncRNAs have been reported to modulate the function of Tregs and promote Treg cell differentiation to create an immunosuppressive state through directly binding to the intracellular domain of membrane receptors. lnc-EGFR was found to induce naive CD4+ T cell precursors to differentiate into Tregs, and the overexpressed lnc-EGFR increased the percentage of Tregs infiltrating in hepatocellular carcinoma and promoted the growth of the tumor. Mechanistically, lnc-EGFR specifically binds to cytoplasmic EGFR and sustains its ubiquitination to trigger the expression of Foxp3, signifying differentiation towards the regulatory T cell lineage (101). Similarly, lnc-INSR was also reported to promote the differentiation of Tregs in pediatric acute lymphoblastic leukemia through enhancing Foxp3 expression via PI3K/AKT by blocking the ubiquitination site of INSR (102). In addition, lncRNA SNHG1 could also promote the differentiation of Treg by suppressing the expression of miR-448, further alleviating the immune escape in breast cancer by elevating the IDO level (103). In a similar case, lncRNA SNHG16 confer Treg-immunosuppressive ability by serving as a ceRNA sponging miR-16-5p; accordingly, the TGF-β1/SMAD5 pathway has been activated and Treg has been motivated (104). By guiding c-MYC to the promoter region, RP11-323N12.5 induces the expression of the transcription factor YAP1, which promotes Treg cell differentiation in gastric cancer (105).

Apart from affecting the differentiation of Tregs, lncRNA could promote the proliferation of Treg, resulting in the expansion of Tregs in the TME. Deregulated lncRNA FENDRR was validated to augment Tregs ratio through enhancing the secretion of immune-related factors in hepatocellular carcinoma cells, such as TGF-β, IL-10, and VEGF that promote the expansion of Tregs (106). Moreover, the upregulation of linc-POU3F3 could promote the differentiation of Treg and further increase the ability of cell proliferation by TGF-β signaling, thus enhancing the growth of gastric cancer (107).

### 5.6 Ths

Th1-polarized CD4+ T cells promote the cytotoxic activities of CTLs and enhance the antigen presentation by antigen-presenting cells (APCs). Contrastingly, Th2-polarized CD4+ T cells are imperative pro-tumorigenic components for tumor cell survival and proliferation (108–110). Emerging evidence suggests roles of lncRNAs in response to the antitumor immune response through decreasing Th1 cell infiltration. Recently, it has been demonstrated that lncRNA SATB2-AS1 could increase Th1 infiltration through upregulating Th1-type chemokine expression such as CXCL9 and CXCL10, thus facilitating immune escape and restraining colorectal cancer progression (111). Contrary to SATB2-AS1, lncRNA HOTTIP could decrease Th1 infiltration through upregulating the expression of the cytomembrane molecule PD-L1 by enhancing STAT-3 activity, thereby accelerating the immune escape of ovarian cancer (84). Additionally, lncRNA SGK1 could intervene the equilibrium between Th1 and Th2 so as to create a microenvironment conducive to immune escape by triggering JunB signaling. The overexpression of lncRNA SGK1 can promote Th2 differentiation while suppressing the differentiation of Th1, thus contributing to the tumor progression of gastric cancer (112).

## 6 lncRNAs and effective tumor-specific T cell-mediated cancer immunity

Antigen binding by MHC coupled with recognition and interaction with the TCR is a critical step for cancer cell elimination mediated by CTLs (113). However, activated antigen-specific CTLs infiltrated in the TME are rapidly anergized, and this dysfunction results in immune evasion and tumor progression.

Increasing evidence indicated that lncRNAs are involved in CTL apoptosis—for instance, lncRNA SNHG14 had been reported to induce CTL apoptosis through upregulating PD-L1 expression in DLBCL cells in order to abstain from immune evasion. The increased SNHG14 augmented the apoptosis proportion of CTLs in TME through promoting the tumor-derived ligand PD-L1. Mechanically, SNHG14 inhibited miR-5590-3p from targeting ZEB1, which upregulated the cytomembrane expression of PD-1 through binding to its promoter, sequentially suppressing DLBCL cell proliferation, invasion, and EMT (114). Analogously, another example is the lncRNA NKILA, which had been identified to significantly upregulate in activated CTLs and sensitized the T cell activation-induced cell death (AICD) by inhibiting NF-κB activity. NKILA silencing declined the sensitiveness of CTL subsets to cell death induced by cancer cells, thus increasing the infiltration density and cytotoxicity of CTLs (115).

Upon chronic exposure to antigens, T cells can develop an exhaustion phenotype in the absence of costimulatory signals or high engagement of inhibitory receptors (116–118). Exhausted T cells are a hallmark of cancer, exhibiting defective proliferation capacities and severely impaired antitumor effector responses.

Recent studies show the direct involvement of lncRNA in T cell exhaustion in such a way that lnc-Tim3 was involved in tumor antigen-activated CTL anergy. lnc-Tim3 was upregulated in tumor-infiltrating CD8+T cells from hepatocellular carcinoma patients, and the antigen-specific CD8+ T cell activity against immunogenic tumors was diminished. The overexpression of lnc-Tim3 contributed to the increase of the rate of exhausted phenotype CTLs by competitively binding to the IC tail of Tim-3, thereby influencing tumor immune evasion (119). These data suggest that the T cell-mediated antitumor immunity can be directly modulated by lncRNAs through interfering with activation-induced cell death or enhancing T cell exhaustion.

In summary, lncRNAs that engaged in reaction to cytokines and stresses that trigger immune reprogramming exhibit highly specific administrative functions that are required for antitumor immunity and tissue homeostasis.

## 7 Diagnosis and therapeutic applications of lncRNA in tumors

lncRNAs are now known to be key regulators for fine-tuning certain aspects of the cancer immunity cycle as well as remodeling of the immune microenvironment. As the expression of lncRNA is tissue- and spatio-temporal-specific, monitoring the changes of immune-related lncRNA expression levels can reflect the current immune microenvironment of tumor cells. Jie Ren (120) performed LASSO-Cox regression analysis and screened four prognosis of lung adenocarcinoma-associated lncRNA. Thereinto, patients who highly expressed CASC15 and LINC01137 have a poor prognosis and are immunosuppressed; however, CRNDE and CYP1B1-AS1 exhibited lower expression levels in lung adenocarcinoma and were closely related to the immunosuppressive microenvironment. Another similar study found that higher expression levels of oncogenic lncRNA AC099850.3, UCA1, and AP005233.2 in pancreatic adenocarcinoma indicate an immunosuppressive state while the protective factors AL513165.1 and PTOV1-AS2 imply an immune status that suppresses cancer (121). Therefore, the detection of immune-related lncRNAs has a huge application prospect in the evaluation of tumor prognosis and immunotherapy efficacy.

Conventional immunotherapy has been developed to initiate a successful antitumor T cell response through strengthening DC ability to prime both CD4+ and CD8+ T cell activation, enhancing T cell trafficking and infiltration into the tumor bed and inhibiting by immunosuppressive cells (TAMs, MDSCs, and Tregs) in the tumor microenvironment (55, 122). Considering the pivotal roles of lncRNAs in cancer immunity, lncRNA-based therapeutics may represent promising approaches to immunotherapy. In breast tumor, treatment with LINK-A locked nucleic acid (LNA) combined with immune checkpoint blockers exhibits a synergistic effect in restraining breast tumor growth (123). lncRNA-directed targeted therapy using LNAs combined with anti-PD-1 immunotherapy reduces the resistance of the latter and serves as a promising strategy to improve triple-negative breast cancer (TNBC) antigenicity and sensitizes breast tumor patients to immunotherapy. NKILA silencing enhances T cells’ resistance to apoptosis and protect the transferred T cells from tumor-mediated AICD. Thus, NKILA may significantly enhance the therapeutic efficiency of adoptive cell therapy (ACT) (115). However, the synergistic effect of lncRNAs with immunotherapy remains marginal.

## 8 Conclusion and future perspectives

lncRNAs are widely involved in modulating the recruitment, expansion, and function of tumor-infiltrating leukocytes, such as DCs, immunoregulatory TAMs, MDSCs, Tregs, and effector T cells such as Ths and CTLs. The versatile mechanisms of lncRNAs in tumor immune escape-associated lymphocytes are listed in **Table 1**, and in consensus with the tissue and disease specificity of lncRNAs (124, 125), the dysregulated lncRNA implicated in tumor escape from immune surveillance might be used to monitor cancer progression and indicate the prognosis of cancer. lncRNAs in the nucleus interact with adjacent genes, acting as a scaffold to recruit chromatin modifiers to enhance or suppress transcription at the transcriptional and epigenetic levels, such as LNMAT1 (35), Lnc-CHOP (95), and NKILA (115). lncRNAs exported to the cytoplasm can interact with specific proteins to influence signaling pathways, modulate the translation of specific mRNAs, or act as microRNA sponges (126)—that is, lncRNAs can modulate mRNA stability by recruiting RNA-binding proteins to degrade mRNA, such that Pvt1 (98) could promote MDSC differentiation via the promotion of c-Myc. lncRNAs can also exert its immunoregulation through interaction with the protein, such that lnc-EGFR (101) and lnc-INSR (102) can directly bind to the cell signal transduction cascades and activate signaling to affect the recruitment and activation of immunosuppressive lymphocytes. In addition, lncRNAs can diminish the miRNA levels by serving either as sponges or by impeding their processing into mature miRNAs, such as SNHG14 in CTLs of diffuse large B cell lymphoma (114) and NIFK-AS1 in TAMs of endometrial cancer (69).

**Table 1:** Long non-coding RNA implicated in tumor immune escape.

lncRNA	Regulation	Localization	Immunity-related molecular mechanisms Reference
lncRNA in antigen presentation			
HOTAIRM1	down	Cytoplasm	Suppress DC differentiation competitively binding to miR- 3960 (24)
lnc-DC	up	Cytoplasm	Promote DC differentiation by binding to STAT3 and sustaining its activation (25)
MALAT1	up	Cytoplasm	Induce the generation of immunosuppressive DC by promoting DC-SIGN expression (20)
NEAT1	up	Nuclear	Induce the generation of immunosuppressive DCs by epigenetically upregulating its target gene (26)
lncRNA in T cell Recruitment			
lncRNA-135528	up	Cytoplasm	Upregulate CXCL10 through the JAK/STAT pathway (30)
JP X	up	Cytoplasm	lncRNA JP X functions as a sponge of miR-378g to upregulate CCL5 (31)
LNMAT1	up	Nuclear	Epigenetically upregulate CCL2 to recruitment TAMs (35)
lnc-BM	up	Cytoplasm	Upregulate CCL2 through the JAK/STAT pathway to recruitment TAMs (36)
JHDM1D-AS1	up	Cytoplasm	Elevate the infiltration of macrophage by upregulating HGF and FGF1 (37)
lnc-sox5	up	Cytoplasm	Restrain CTL infiltration by activating IDO1 (39)
XIST	up	Cytoplasm	Promote the recruitment of MDSCs by sponging miR-133a-3p (40)
LINC01094	up	Nuclear	Promote the recruitment of macrophage by upregulating CCL7 (38)
lncRNA in T cell trafficking			
ZFAS1	up	Cytoplasm	Upregulate VEGFA as ceRNA to sponge miR-150-5p (44)
H19	down	Cytoplasm	Suppress the expression of adhesion molecular repressing via activation of STAT3 (46)
AF131217.1	up	Cytoplasm	Suppress the expression of adhesion molecular by sponging miR-128-3p (47)
lncRNA in Tregs			
lnc-EGFR	up	Cytoplasm	Promote Treg differentiation by binding to EGFR (101)
lnc-INSR	up	Cytoplasm	Promote Treg infiltration by binding to INSR and sustained its activation (102)
FENDRR	down	Cytoplasm	Suppress Treg function by sponging miR-423-5p (106)
Linc-POU3F3	down	Cytoplasm	Promote Treg infiltration recruit by activating the TGF-β signal pathway (107)
SNHG16	up	Cytoplasm	Promote Treg differentiation by sponging miR-16-5P (104)
RP11-323 N12.5	up	Nuclear	Promotes Treg cell differentiation by guiding c-MYC to the promoter region and inducing the expression of the transcription factor YAP1 (105)
lncRNA in MDSCs			
Lnc-CHOP	up	Nuclear	Promote MDSC differentiation and function as scaffold of LIP/CHOP complexes (95)
RUNXOR	up	Cytoplasm	Promote MDSCs function and infiltration by downregulating RUNX1 expression (99)
Pvt1	up	Cytoplasm	Promote MDSC differentiation and function via promotion of c-Myc stability (98)
HOTAIRM1	down	Cytoplasm	Promote MDSC function and infiltration by promoting the expression of HOXA1 (20)
Lnc57Rik	up	Nuclear	Promote MDSC differentiation and function as scaffold of LIP/CHOP complexes and help WDR5 to achieve methylation modification function (96)
Snhg6	up	Cytoplasm	Promote MDSC differentiation and function via inhibiting the expression of EZH2 by means of ubiquitination pathway (100)
lncRNA in CTLs			
lnc-Tim3	up	Cytoplasm	Promote CTL exhaustion by binding to Tim-3 (119)
SNHG14	up	Cytoplasm	Upregulate PD-L1 and induce CTL apoptosis by sponging miR-5590-3p (114)
NKILA	up	Cytoplasm	Induce activation-induced cell death of CTLs by inhibiting NF-κB activity (115)
lncRNA in Ths			
SATB2-AS1	down	Nuclear	Recruit H3K4me3 SATB2-AS1 and cis-activate SATB2 to suppress Th1 (111)
HOTTIP	down	Cytoplasm	Decrease Th1 infiltration and restrain Th1 function by bind to c-jun (84)
Lnc-SGK1	up	Nuclear	Promote Th2 polarization by cis-activated SGK1 expression (112)
lncRNA in TAMs			
UCA1	up	Cytoplasm	Promote cancer progression induced by TAMs to activate the AKT signaling pathway (61)
MALAT-1	up	Nuclear	Inhibit immunosuppressive cytokine secretion by upregulating FGF2 expression (60), synergize with HOTAIR to inhibit the expression of CD80 and MLSN (62)
HOTAIR	up	–	Inhibit M1-like macrophage polarization, synergizes with MALAT-1 to promote cancer (62)
CCAT1	down	Cytoplasm	Restrain M2 polarization by sponging miR-148a to upregulate PKCζ (68)
NIFK-AS1	down	Cytoplasm	Restrain M2 polarization by sponging miR-146a to upregulate Notch1 (69)
cox-2	up	both	Promote M2 polarization (64)
GNAS-AS1	up	Cytoplasm	Promote M2 polarization by sponging miR-4319 to promote NECAB3 expression (63)
LINC00543	up	Cytoplasm	Promote M2 polarization by sponging pre-miR-506-3p (65)
LINC00313	up	Cytoplasm	Promote M2 polarization by upregulating STAT6 (74)
lncRNA-PA CERR	up	Cytoplasm	Promote the differentiation of M2-like macrophages via enhancing the mRNA stability of KLF12 and c-myc by binding to IGF2BP2 (67)
PACERR		Promote M2 polarization by upregulating PTGS2 (67)
LARRPM	down	Nuclear	Restrain M2 polarization by guiding TET1 to the CSF promoter (70)
LINC00702	down	Nuclear	Restrain M2 polarization by impairing DUSP1 transcription through recruiting JUND to its promoter (71)
HOTTIP	down	Cytoplasm	Promote M1 polarization by targeting miR-19a-3p and miR-19b-3p, thereby activating the TLR5/NF-κB signaling pathway
lncRNA in TANs			
LINC01116	up	Nuclear	Promote neutrophil recruitment by upregulating IL-1β (82)
MIR443 5-2HG	up	–	Promote N2 phenotype neutrophil recruitment and infiltration (83)
HOTTIP	up	Nuclear	Promote N2 phenotype neutrophil-mediated CTL apoptosis via IL-6 signaling pathway (84)
lncRNA in NKs			
SNHG10	up	Cytoplasm	Restrain the cytotoxicity of NK cells by activating TGF-β signaling pathway (86)
NCAL1	down	Nuclear	Promote the cytotoxicity of NK cells by increasing Gab2 promoter H3K4me3 and H3K27ac levels (87)
GAS5	down	Cytoplasm	Promote the cytotoxicity of NK cells by sponging mir-18a (88)
EPB41 L4A-AS1	up	–	Restrain the cytotoxicity of NK cells by damaging the glycolysis process (89)
MALAT-1	up	–	Restrain the cytotoxicity of NK cells by inducing apoptosis mediated immune checkpoints (90)

Although much progress has been made in cataloging lncRNAs expressed in cancer immunity, there remains a large void before their effective use as therapeutic targets. Firstly, a considerable amount of research is needed to examine the toxicity and the pharmacokinetics of lncRNA-based therapeutics, such as ASOs. Secondly, lncRNAs generally demonstrate poor evolutionary sequence conservation across species (127), thereby preventing the use of a traditional approach for the identification of its function. Thirdly, it is difficult to identify the downstream targets of lncRNA because the presently available bioinformatics tools for lncRNA target prediction are far from precisely identifying the functional lncRNA motifs and structure. This underlines the need for novel techniques directed towards predicting and demonstrating the structure of lncRNAs, dictating the localization and interactions of these molecules (128–130). Additionally, new methods for addressing the impact of lncRNAs on higher-order 3D chromatin organization and epigenomic regulation analysis of lncRNA in cancer subpopulations or single cell will further facilitate the study of lncRNAs in primary cancer tissues (131–133). Therefore, the clinical application of lncRNAs merits further investigations in the future.

## Author contributions

D-tZ wrote and reviewed the original draft. H-cX reviewed and edited the manuscript. All authors contributed to the article and approved the submitted version.
